# Progress of Dicyanomethylene-4H-Pyran Derivatives in Biological Sensing Based on ICT Effect

**DOI:** 10.3389/fchem.2022.903253

**Published:** 2022-05-23

**Authors:** Ting-Ting Hou, Yi Cai, Zhen-Yu Zhang, Cai-Yun Wang, Ying-Hao Tang, Ming-Qiang Zhu, Ya-Long Wang

**Affiliations:** ^1^ Key Laboratory of Biomedical Engineering of Hainan Province, School of Biomedical Engineering, Hainan University, Haikou, China; ^2^ Wuhan National Laboratory for Optoelectronics, Huazhong University of Science and Technology, Wuhan, China; ^3^ One Health Institute, Hainan University, Haikou, China

**Keywords:** dicyanomethylene-4H-pyran (DCM), intramolecular charge transfer, near-infrared probe, bioimaging, biosensor

## Abstract

As one of the typical fluorescent cores, dicyanomethylene-4H-pyran (DCM) derivatives exhibit excellent photophysical and photochemical properties, such as large Stokes shift, excellent light stability, and tunable near-infrared (NIR) emission. The luminescence mechanism of DCM probes mainly depends on the intramolecular charge transfer (ICT). Hence, by regulating the ICT process, the probes can specifically act on the target molecule. Accordingly, a series of NIR DCM probes have been constructed to detect the ions, reactive oxygen species (ROS), and biological macromolecules in cells. However, there is no relevant review to summarize it at present. This minireview mainly summarizes the NIR DCM probes based on ICT effect and their applications in biosensors and biological imaging in recent years. This will be beneficial to innovatively construct new DCM probes and actively promote their application in the future.

## Introduction

Dicyanomethylene-4H-pyran (DCM) is a typical fluorophore, which shows excellent photophysical and photochemical properties, such as large Stokes shift, excellent light stability, and tunable near-infrared emission (Wang et al., 2016; Nabavi et al., 2018). Derivatives derived from DCM have been widely applied in nonlinear optical materials (Guo et al., 2012), logic gates, photovoltaic sensitization (Liu et al., 2009), sensing, and other fields. Compared with cyanine dyes, the excellent photophysical and photochemical properties of DCM derivatives are much conducive to the application in real-time evaluation, detection of analytes, and long-term tracking imaging (Chao et al., 2019). Moreover, the emission peaks of DCM derivatives are usually located at >600 nm, which makes the compounds easily excited by near-infrared (NIR) after simple modification. Dyes with NIR emission are beneficial for biological imaging due to their deep tissue penetration, weak background interference, and negligible cell damage (Guo et al., 2014). Furthermore, DCM dyes possess the merits of high quantum yield, two-photon absorption cross section (Nawimanage et al., 2017; Luo et al., 2021), and easy synthesis methods (Li et al., 2014; Li et al., 2018). Therefore, DCM probes are regarded as promising candidates for biosensors and biological imaging.

**FIGURE 1:**
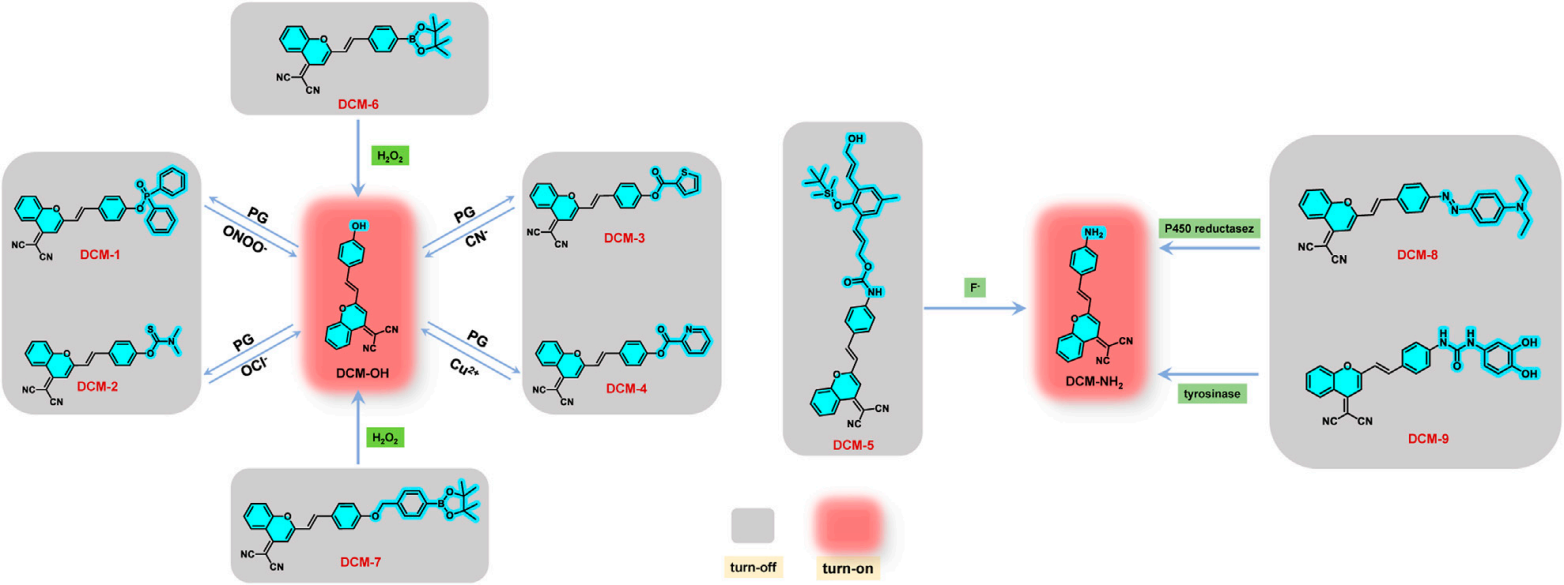
Structure and mechanism of DCM-1∼9 for detection of different substances.

Generally, DCM molecules possess a D-π-A configuration, showing a typical ICT effect. ICT effect usually occurs in a molecule with a D-A structure. When the probe with the ICT effect is activated, the charge is apt to flow from the donor (D) segment to the acceptor (A) segment, resulting in the variation of luminescence. Hence, the emission properties of DCM probes can be adjusted as required by tuning the molecular conjugation system or altering the electron donor group. In addition, DCM probes with an “off–on” function have been obtained successfully by introducing fluorescence-quenching groups as well as other active groups. When the probe contacts the target, the ICT mechanism of the DCM probe is reactivated due to the separation of the fluorescence-quenching group by reaction with the target (Figure 1). Thus, the probe lights up afresh. Based on this principle, a series of “off–on” DCM derivatives have been successfully constructed for the detection of ions, reactive oxygen species (ROS), and biological macromolecules in cells (Li et al., 2014; Wang et al., 2019; Yang et al., 2019; Dong et al., 2020; Ling et al., 2021). Although much attention has been paid on this kind of probes in recent years, a systematic overview is rarely reported. Recent advances in “off–on” DCM probes based on the ICT mechanism and their applications are highlighted. The current challenges for large-scale applications are discussed as well.

## Applications

DCM derivatives with “off–on” characteristics have been successfully applied in detection of ions, hydrogen peroxide, and enzymes. In this section, we will systematically and roundly discuss the current applications of “off–on”-type DCM derivatives with NIR emission based on the ICT mechanism.

### Detection of Ions

**FIGURE 2:**
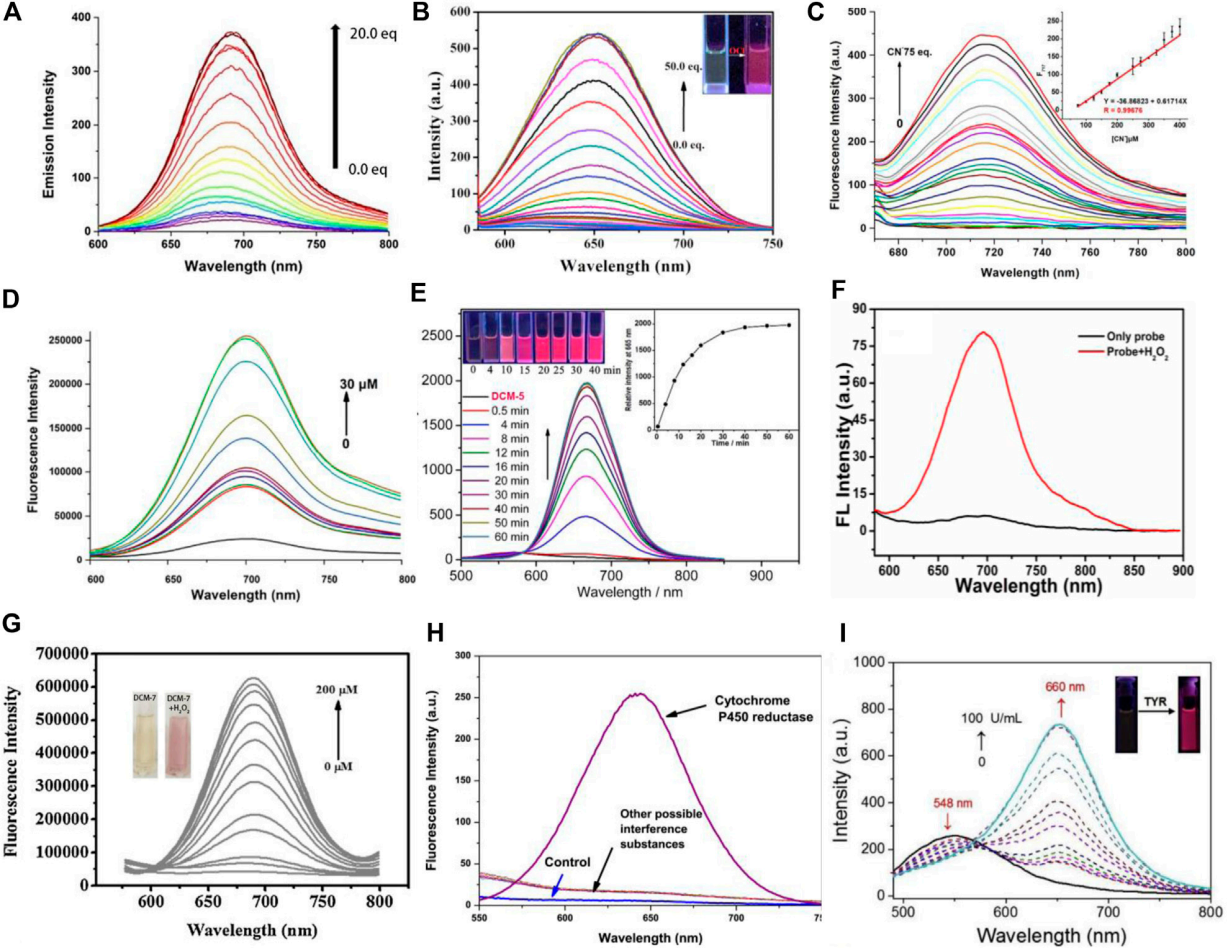
**(A)** Fluorescence emission spectral changes of DCM-1 with various concentrations of ONOO^−^ in solution. **(B)** Fluorescence emission spectra of DCM-2 upon gradual addition of OCl^−^ in EtOH: H_2_O = 1:1 (v/v). **(C)** Fluorescence emission spectra of DCM-3 upon the addition of CN^−^. **(D)** Fluorescence spectra of DCM-4 with different concentrations of Cu^2+^ in PBS solution. **(E)** Time-dependent emission spectra of DCM-5 with F^−^ in aqueous solution. **(F)** Fluorescence spectra of DCM-6 in the presence of H_2_O_2_ with excitation at 560 nm. **(G)** Fluorescence spectra measurement of DCM-7 in the presence of different concentrations of H_2_O_2_. **(H)** Specific detection of cytochrome P450 reductase among various reductants with DCM-8. **(I)** Emission spectra of DCM-9 with TYR in aqueous solution.

Ions are closely related to life activities and play a vital role in the field of life (Gale et al., 2008). Therefore, it is of great significance to develop fluorescent probes with excellent recognition for ions. Based on DCM-OH, a series of NIR probes DCM-1∼4 was designed and synthesized by introducing various ester-protecting groups (PG) as well as fluorescence-quenching groups (Figure 1). In the presence of specific ions, the PG is liable to detach, activating the ICT effect to emit fluorescence (Figure 1). Mulay et al. (2017) reported a NIR probe DCM-1 based on DCM, showing remarkable sensitivity to ONOO^−^. The probe was prequenched skillfully with the diphenyl hypophosphite group, which was sensitive to ONOO^−^ (Figure 1). The fluorescence of DCM-1 turned on again in the presence of ONOO^−^ in a few minutes with emission enhancement of 120-fold, indicating high selectivity and sensitivity (Figure 2A). Moreover, DCM-1 can also track ONOO^−^ in cells. By replacing the fluorescence-quenching group with dimethyl thiocarbamate (DMTC), Zhang et al. (2019) designed and synthesized an OCl^−^-targeting probe DCM-2 based on the DCM core (Figure 1). DCM-2 could rapidly generate fluorescence response to OCl^−^ ions within 3 s, with a low detection limit of 80 nM, behaving with high selectivity and sensitivity to OCl^−^ (Figure 2B). Furthermore, the probe was successfully applied in detecting endogenous/exogenous OCl^−^ in living cells. Those works provide inspirations for designing other ion sensors based on the DCM skeleton.

Recently, Wang et al. (2020) and Li et al. (2021) reported two DCM-type probes DCM-3 and DCM-4 by replacing the protecting group (PG) (Figure 1). DCM-3 exhibited remarkable selectivity and sensitivity to CN^−^, with the detection limit of 1.44 μM (Figure 2C). DCM-4 emitted NIR fluorescence when Cu^2+^ traces were added, with the detection limit of 25.4 nM (Figure 2D). Further studies showed that DCM-4 was insensitive to other ions. Those results demonstrate that DCM-4 possesses satisfactory sensitivity and selectivity toward Cu^2+^. Furthermore, the MTT assay showed that DCM-4 possesses low cytotoxicity and excellent biocompatibility. In addition, a probe DCM-5 sensitive to F^−^ was reported by Feng et al. (2019) based on the DCM derivative DCM-NH_2_. A Si-O-connected detachable group was introduced as a fluorescence quenchant, and a specific fluoride trigger was elaborately designed (Figure 2E). The fluorescence was activated after the addition of F^−^ in several minutes with a detection limit as low as 157 nM, displaying high sensitivity and selectivity of F^−^. Further studies showed that DCM-5 can be successfully applied to the quantitative detection of exogenous fluoride in HeLa cells and zebrafish embryos by fluorescence imaging. Those research studies not only expand the application of DCM-type probes but also provide strategies for the development of subsequent probes.

### Detection of Hydrogen Peroxide

Hydrogen peroxide is a kind of reactive oxygen species (ROS), which plays a vital role in a variety of physiological processes. Studies have shown that the imbalance of H_2_O_2_ is associated with cardiovascular disease, neurodegenerative disease, Alzheimer’s disease, cancer, and other serious diseases (Wang et al., 2015; Li et al., 2017; Zhou et al., 2019). Therefore, it is of great significance to develop probes to detect the concentration of hydrogen peroxide in cells.

By introducing borate ester groups sensitive to H_2_O_2_, DCM-6∼7 was obtained with fluorescence quenching because the electron-donating effect of -OH was blocked (Wang et al., 2015; Li et al., 2017; Zhou et al., 2019). In the presence of H_2_O_2_, the fluorescence of DCM-6 and DCM-7 is turned on due to the restoration of the ICT effect *in vivo* and *in vitro*, showing excellent sensitivity and selectivity toward H_2_O_2_ (Figures 2F, G). It is worth mentioning that DCM-7 showed dual signal with colorimetry and fluorescence in response to H_2_O_2_ (Figure 2G), achieving more reliable detection results. The successful tracks of exogenous and endogenous H_2_O_2_ in living cells suggest great potential in bioassay.

### Detection of Enzymes

The enzyme, a kind of biological macromolecule, is one of the indispensable substances in vital activities. Almost all kinds of reactions in organisms cannot be separated from the catalysis of enzymes. In addition, enzymes are closely related to the occurrence and development of some diseases. Therefore, effective labeling and detection of enzymes have aroused widespread concerns. Cui et al. (2017) and Li et al. (2019) designed and synthesized DCM-8 and DCM-9 based on DCM-NH_2_, respectively (Figure 1). DCM-8, an off–on probe that is sensitive to cytochrome P450 reductase, was quenched with the azo group. After reacting with cytochrome P450 reductase, NIR fluorescence turned on due to the generation of DCM-NH_2_ (Figure 2H). The fluorescence intensity was strengthened by more than 156 times after reacting with cytochrome P450 reductase and NADH system for 4 min. Moreover, the probe exhibited strong antiinterference ability and high sensitivity. Tyrosinase (TYR) is a significant biomarker of melanoma cancer and plays an important role in cellular biochemistry and etiology (Lin et al., 2016). Li et al. (2019) reported NIR probe DCM-9 for the detection of endogenous tyrosinase. In the presence of TYR, the tyrosinase trigger group escaped from the DCM core, generating NIR dye DCM-NH_2_. DCM-9 possessed high sensitivity and selectivity toward TYR (Figure 2I). The MTT assay demonstrated that DCM-9 exhibits low cytotoxicity and good biocompatibility for B16 melanoma cells. Furthermore, the probe was successfully applied to the fluorescence imaging of endogenous TYR in B16 melanoma cells. Thus, the enzyme-activatable NIR probe based on DCM may be a powerful tool for investigating the important roles of enzymes in biological systems.

## Conclusion

To sum up, DCM derivatives modified with fluorescence-quenching group have shown typical off–on characteristics. According to the different activated substances, the dyes are rationally divided into three sections in this minireview. They are potential candidates for biosensing due to their rich structures and conformations. DCM derivatives have broad prospects in biosensing and bioimaging due to their numerous merits. In the future, more DCM probes will be designed, and efforts focus on the following aspects: 1) design of new-type DCM derivatives with water-solubility, which exhibit great potential in detection of physiological environment, bioimaging, and disease detection drug carriers, etc., 2) developing more applications for DCM probes, such as new iron detection, detection of important active enzymes in the human body, disease screening, and visualization of latent fingerprints. In short, this mini minireview has summarized most of the excellent reports of off–on DCM probes with red emission in this field. It is anticipated that DCM probes with fluorescence activation features will attract more and more interest and greatly flourish.
